# Facilitating Automated Data Analytics Through Structured Head and Neck Oncology Tumor Board Documentation

**DOI:** 10.1200/CCI.21.00168

**Published:** 2022-03-08

**Authors:** Pratyusha Yalamanchi, Keith A. Casper, Joseph Evans, Michelle Mierzwa, Robert J. Morrison, Mark E. Prince, Charles S. Mayo

**Affiliations:** Pratyusha Yalamanchi, MD, MBA, and Keith A. Casper, MD, Department of Otolaryngology-Head and Neck Surgery, Michigan Medicine, Ann Arbor, MI; Joseph Evans, MD, and Michelle Mierzwa, MD, Department of Radiation Oncology, University of Michigan, Ann Arbor, MI; Robert J. Morrison, MD, and Mark E. Prince, MD, Department of Otolaryngology-Head and Neck Surgery, Michigan Medicine, Ann Arbor, MI; and Charles S. Mayo, PhD, Department of Radiation Oncology, University of Michigan, Ann Arbor, MI

## TO THE EDITOR:

Multidisciplinary tumor boards (TBs) have increasingly become the standard of care for developing optimal treatment plans for complex cancer patients.^1-3^ Head and neck cancer treatment frequently involves multidisciplinary care, highlighting the importance of a comprehensive TB. TBs have been shown to improve patient outcomes and enhance high-quality patient care, resulting in altered treatment plans in up to 50% of reviewed cases and improved survival for solid organ tumors.^1-5^ Weekly TB preparation compiles clinically relevant data from a variety of sources and has been shown to be both time- and labor-intensive.^6^ When TB data are subsequently used for institutional quality improvement efforts or clinical research such as traditional retrospective analysis, repeated manual chart review is typically required, resulting in additional, time-consuming effort prone to human error. Here, we present a simplified, expert-driven TB documentation workflow that automates oncologic data capture and reporting. This provides a framework for regular sharing and analysis of treatment-related outcomes and quality metrics and a method for efficient clinical trial screening and recruitment.

### University of Michigan Multidisciplinary Head and Neck Oncology Tumor Board

Multidisciplinary head and neck TB conferences occur weekly at our institution in a virtual format led by a head and neck surgeon and last approximately 2 hours. Quorum requires, at a minimum, at least one faculty member from head and neck surgery, medical oncology, radiation oncology, head and neck neuroradiology, and nuclear medicine. Before each weekly conference, cases are submitted throughout the prior week, and internal and external pathologic slides and diagnostic imaging are reviewed by attending-level specialists. Structured TB notes that collate relevant clinical data are prepared in advance by resident physicians for presentation at the conference. Treatment recommendations, disease status, and clinical trial candidacy are then recorded during the meeting by the head and neck oncology fellow on the basis of group consensus achieved through discussion and recommendations consistent with National Comprehensive Cancer Network (NCCN) guidelines. In the occasional setting of differing opinions or differing treatment recommendations, these are recorded as well; typically, free text is used to elaborate on the differing recommendations.

### Streamlined TB Documentation Workflow

In 2018, our electronic health record Head and Neck Tumor Board template was transitioned to a simplified, expert-designed template with relevant clinical data populated into structured fields with a combination of automatic population from the electronic health record and manual selection of drop-down menus. Before this intervention, all patients discussed at the conference had free text TB documentation recorded either by faculty or resident, inclusive of any relevant information presented at the multidisciplinary meeting. The impetus of this change was to provide a more streamlined documentation and presentation workflow and to facilitate department-specific, user-friendly decision support tools for retrospective review of performance and quality improvement metrics. Four different templates are currently used, titled HNTBPRETX for primary treatment discussion, HNTBPOSTOP for adjuvant treatment decision making, HNTBRECURRENCE for evaluation and discussion of possible recurrent or persistent disease, and HNTBTEMPLATE for case considerations that do not fit into the previously listed categories.

Required, structured fields within each documentation template with associated branching logic ensure uniform, complete data entry regarding disease stage, status, and prior primary and adjuvant therapy (Table 1). Treatment recommendations, disease status, and clinical trial candidacy recorded during the meeting are also populated within structured fields to increase ease of data analyses. Although templates were designed to limit free text to ensure consistent and standardized data entry, free text fields such as Brief Clinical Scenario are available for manual entry of additional relevant information and to facilitate succinct discussion during meetings.

**TABLE 1.**
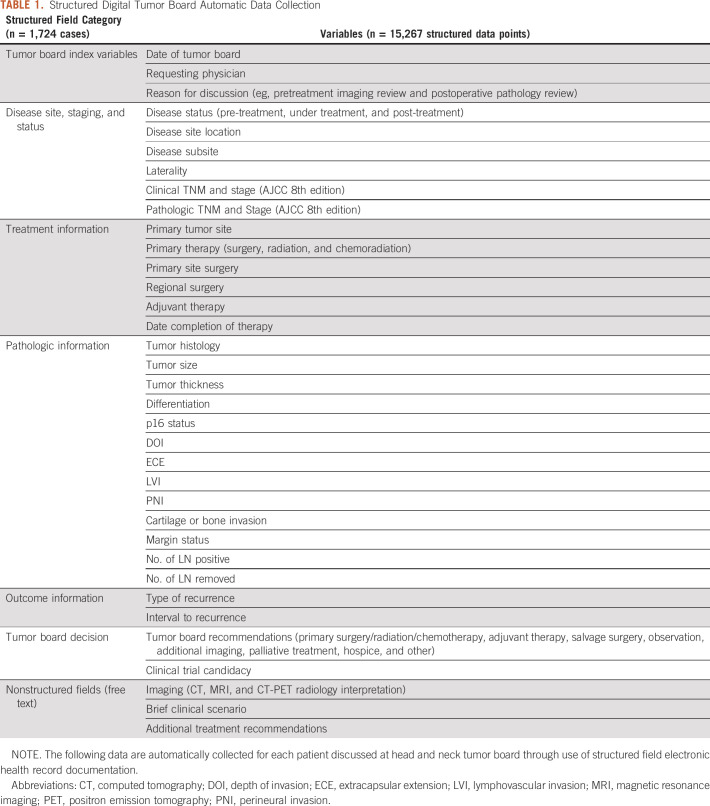
Structured Digital Tumor Board Automatic Data Collection

### Current Clinical Applications of Streamlined TB Documentation Data

**FIG 1.**
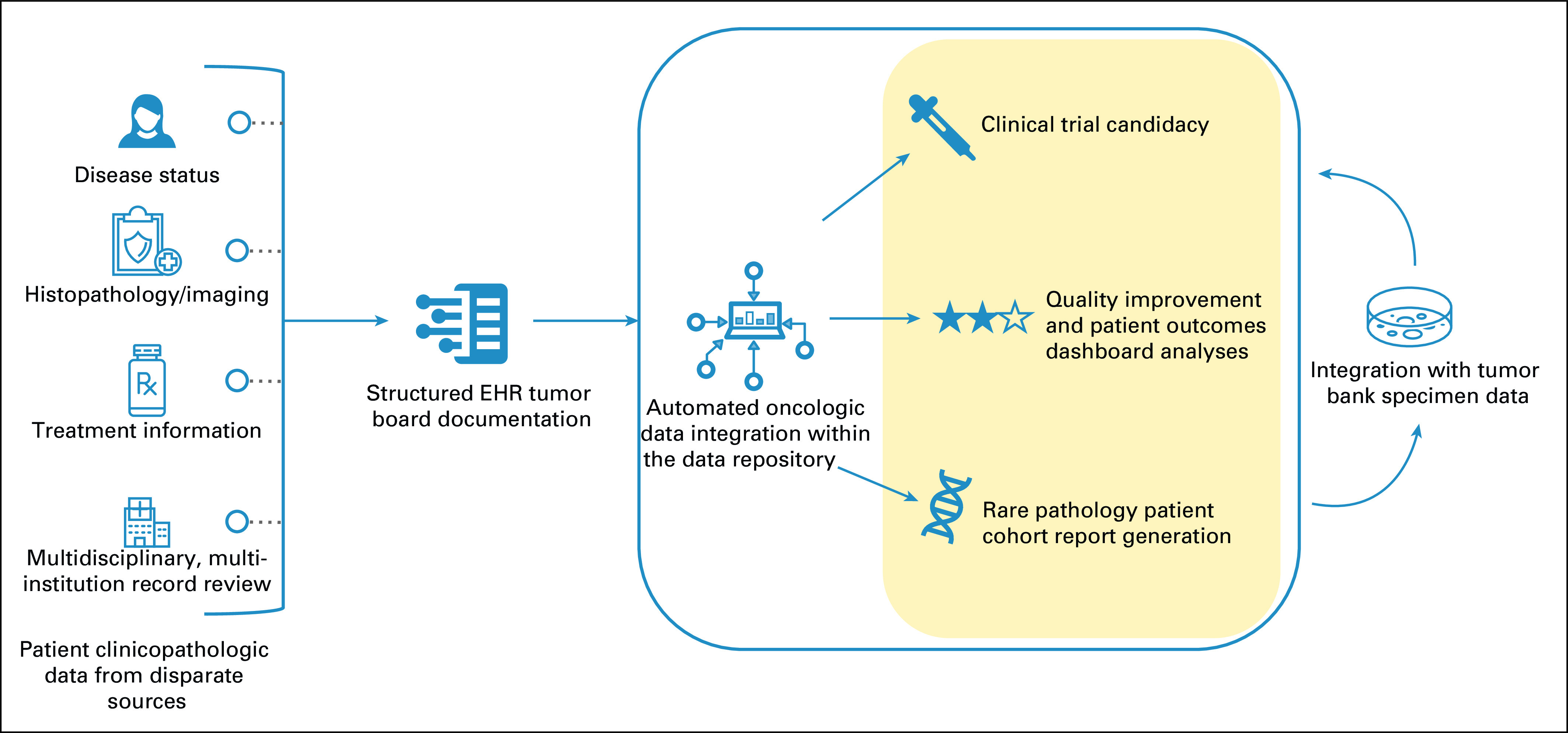
The study flow diagram demonstrates automatic integration of head and neck oncology patient data within a digital tumor board data repository through simplified, standardized EHR documentation, and associated current clinical applications. EHR, electronic health record.

Implementation of this efficient, high-quality documentation workflow in more than 1,700 head and neck TB cases has ensured standardization of processes for case discussion and reduced errors during retrospective analyses performed for clinical research and quality improvement efforts. This simple digital tumor board system built directly into the clinical note has supported the complex workflows of clinical practice, including identification of an additional 162 (9.3%) patients as potential candidates for clinical trials on the basis of categorical inclusion criteria and generating reports of surgical pathology outcomes such as lymph node yield from neck dissection and margin status (Fig 1). Ultimately, this streamlined documentation workflow has facilitated automation of collection, aggregation, and visualization of head and neck oncologic patient data for performance analysis, clinical research, and quality improvement work, and serves as a model that can be easily adapted across disease sites or institutions.
